# A Neural Network Model for Digitizing Enterprise Carbon Assets Based on Multimodal Knowledge Mapping

**DOI:** 10.1155/2022/4485168

**Published:** 2022-06-20

**Authors:** Jiexian Liu, Chen Zhang

**Affiliations:** ^1^School of Management, Hefei University of Technology, Hefei, Anhui 230009, China; ^2^School of Accounting, Tongling University, Tongling, Anhui 244061, China

## Abstract

In this paper, a multimodal knowledge mapping approach is used to digitize enterprise carbon assets, and a corresponding neural network model is designed for use in the practical process. Rich textual entity labels associated with images are obtained using an entity annotation system. A topology-based data fusion method is also designed based on the hierarchical relationship between WordNet and DBpedia to fuse the knowledge obtained from image visualization and text description mining. Existing neural network-based entity linking methods ignore the semantic gap between the context of sequential entity denotative items and the context of graph-structured entities, thus affecting the accuracy of entity linking. It is observed that the importance of words in the context of entity denotative items is different, and the importance of content in the entity context is also different. To solve the above problems, this paper proposes an entity linking method that combines a common attention mechanism with a graph convolutional neural network. Secondly, based on the basic theory of value assessment, the characteristics of classical asset valuation methods and their inapplicability to the valuation of carbon assets are analyzed, and thus the real option valuation method and its two classical models are introduced; after demonstrating the real option characteristics of carbon assets of power enterprise projects, a real option model-based carbon asset valuation model for power enterprise projects is constructed and its applicability is verified with case studies. Through analyzing the current situation and problems of carbon asset valuation work in power enterprises, targeted practical suggestions are put forward to further strengthen and enhance the carbon asset valuation work in power enterprises in the future.

## 1. Introduction

Knowledge graph (KG) provides an efficient data organization and management model and has become a hot research issue in recent years, and its related technologies and applications have made great progress. Currently, two important types of knowledge graph construction are open-domain knowledge graph (GKG) construction and vertical domain knowledge graph (DKG) construction [1]. The data in open domain knowledge graph construction mainly comes from major knowledge bases and search websites and is built in a top-down manner, through entity analysis and extraction of encyclopedic knowledge, and ultimately results in a usable open knowledge network. Already carried out step by step from point to point, from single to multiple, from centralized to comprehensive, it is bound to grow from the most promising carbon asset trading market to the most active carbon trading market, and therefore a scientific and effective value assessment of carbon assets becomes an objective need for the carbon asset trading market to continue to prosper. From the perspective of asset valuation, existing research on carbon assets has focused on the pricing of carbon assets, the initial allocation of carbon assets, and the accounting recognition and measurement of carbon assets [2]. A few scholars have begun to focus their attention on carbon asset management and development [3]. However, there is still no authoritative conclusion on what kind of valuation method can be adopted to assess the value of carbon assets scientifically and accurately, especially the research on the valuation of carbon assets of power enterprises, the main force of the carbon trading market, which can be collected. The traditional market approach, the income approach, and the cost approach are all limited and inapplicable to the valuation of carbon assets and project carbon assets of power enterprises [4].

The market-based approach to carbon emissions has become a major tool for countries around the world to address the climate issues and control pollutant emissions. As the core of the carbon financial market, an effective carbon financial asset pricing mechanism will help promote the maturity and improvement of the carbon financial market mechanism and market efficiency, and better serve the implementation of carbon emission reduction. Compared to the other mature capital markets, the carbon finance market is, on the one hand, vulnerable to the irrational behavior of non-fully rational investors due to its weak market efficiency. On the other hand, it is extremely sensitive to policy dependence [5]. Carbon assets are allowances emission rights, emission reduction credits and related activities that can directly or indirectly affect an organisation's greenhouse gas emissions generated under a mandatory carbon trading mechanism or voluntary carbon trading mechanism. Energy policies, carbon tax policies, quota information, as well as international negotiations and financial crises can all trigger dramatic fluctuations and shocks in carbon finance market prices. Existing research on carbon asset pricing has mainly focused on the lower order moment properties of returns (mean and variance) to study the information transmission and volatility spillover of pricing factors on carbon prices, while neglecting to study the impact of asymmetric market information and extreme event shocks on carbon prices from the perspective of higher-order moment properties (market skewness and kurtosis), a shortcoming that makes the accuracy of existing research on carbon asset pricing questionable [6]. Price risk from market asymmetric information and extreme event shocks within the carbon financial markets can be easily transmitted to other financial or energy markets through global financial networks and, conversely, can be affected by price risk in these markets, i.e., the risk contagion relationship between carbon financial assets and their pricing factors due to shocks in higher-order moment properties can also affect the pricing of carbon financial assets [7].

The risk contagion relationship between carbon financial markets and their pricing factor markets due to higher-order moment properties is analyzed. Based on the risk contagion theory of financial assets, the risk contagion relationship between carbon prices and their pricing factors, especially the risk contagion relationship due to asymmetric information and extreme events, is more in line with the special volatility characteristics of carbon financial market prices; identify the risk contagion channels of the higher-order moments of carbon financial assets; explore the differences in the degree of risk contagion between different market volatility trends, and provide a basis for conducting targeted price studies. The market approach is to use the recent transaction prices of similar or comparable assets in the market to estimate the value of the assets after direct comparison or analogous analysis. The valuation information in the process of applying this method is derived from the market and the valuation results can also be tested by the market, which is in line with the general principles of market economy and easily accepted by both parties to the transaction. The market approach is the most direct, simple, and effective method in asset valuation. This will provide a basis for conducting targeted price studies. Based on a pricing framework for carbon financial assets that takes into account the risk contagion of higher-order moments, the study investigates the impact of the risk contagion of higher-order moments on carbon prices and the explanation of premiums; establishes a nonlinear relationship between carbon prices and the risk contagion of higher-order moments between pricing factors, and explores the intrinsic link between the risk contagion of higher-order moments and carbon financial asset prices based on cross-market linkage theory. This study uses a long- and short-term memory neural network with a long memory function for time series to fit the pricing framework; determines network characteristics such as the number of layers and neuron structure based on experimental means; conducts prediction analysis and model performance evaluation on carbon financial asset pricing models with different higher-order moment risk contagion relationships and carbon prices of different maturities; compares the explanatory power of carbon financial asset pricing framework with and without higher-order moment risk contagion.

## 2. Related Works

A typical example is a solution designed by Riadi et al. to address the lack of multimedia information in DBpedia [8]. They use the concept of entities in DBpedia as keywords in a search engine to search the Internet and define a reasonable filtering mechanism to filter the search results, treating the results as multimedia information extended on top of DBpedia [9]. The main goal of this type of approach is to structure the processing of image metadata so that the association with graphs having knowledge graphs does not occupy a large proportion of the overall dataset. The approach proposed in this paper should be subordinated to the second category, differing more in that it treats the association of image data with the graphical open knowledge graph resources in a significant way [10]. The different methods of determining the commonality of the data in this paper will only affect the number of results and will not have any impact on the structure of the results. Therefore, the method used to determine the commonality of the data by manually constructing a conceptual lexicon is more concerned with the value of the result based on its specific structure rather than the number of images or other resources in the result [11]. The keywords supported by Gallery pages are divided into two types, the first being textual entity concepts and the second being image names. The content of the pages locked by the two types of keywords is also different [12]. The pages locked by the entity concept are mainly a list of images related to the current topic by type, without any more detailed information about the images (name, description, etc.), and each image is bound to a URL, which is a unique page for each image, the same as the page locked by the image name, in this page, the image name, description, type information, etc. are available.

The HTML document to be processed is first divided into different paragraphs, each containing a text area and an image area, according to the characteristics of Wikipedia editing. The text area is the content of the body and is used to develop textual entities that may be associated with the information described in the image. The image area contains two main components: the image itself, which contains visual information, and the caption, which portrays the main semantic information contained in the image in the form of natural language. In the process of dividing the Wikipedia data, the same markers are added to text and image regions belonging to the same paragraph, and after processing them separately by different methods, associations are established between the text and image entities with the same markers [13]. The results show that the method, combined with the market instability index, is effective for early warning of stock market risk levels. This approach allows appropriate policy actions to be taken in response to possible financial crises based on the instability of different risk warning levels.

Typically, there is a positive correlation between the price of carbon and the price of cars. A detailed study of carbon trading prices was conducted using RS analysis, which concluded that the factors such as the economic environment, allowance allocation system, and policy preferences have a strong influence on the price of carbon trading. Therefore, focusing on changes in the economy, allowances, and policies are beneficial to the prediction of prices. Using the VAR model and VECM model, different instruments were adopted and relevant data from the Shenzhen carbon emission rights exchange were collected to comprehensively analyze the influencing factors of carbon prices [14]. After systematic analysis, it was found that the most influential factor on the domestic carbon emission rights trading price is the coal price, therefore, the carbon emission rights trading price should focus on the coal price. After considering the impact of fossil energy on the price of carbon emissions trading from other perspectives, it was found that the changes in the price of fossil energy directly or indirectly affect the change in the price of carbon emissions trading, and there is a positive relationship between them. Changes in the price of coal can cause changes in the price of carbon credits. Changes in the price of oil also have an impact on changes in the price of carbon trading, with the former being greater than the latter.

## 3. Analysis of a Digital Neural Network Model for Carbon Assets of Multimodal Knowledge Mapping Enterprises

### 3.1. Digital Multimodal Knowledge Mapping Design

The goal of multimodal knowledge extraction is to extract the target knowledge given multi-source heterogeneous data using manual or automated methods such as machine learning and deep learning. Manual extraction generally uses experts or organized discussions and requires more human resources. Automated extraction, however, is based on the rapid development of computer technology and has produced many results in recent years, from the early days of textual knowledge extraction to the extraction of modally diverse data such as images, audio, and video. Based on the current state of research, knowledge extraction methods can be divided into entity extraction, relationship extraction, and attribute extraction. The results obtained are used to learn from the labeled images involved to obtain a training model, which can continuously update the entities using the recognition of unfamiliar images.

(1) $$\begin{array}{c} P\left(y|x\right)=\frac{1}{z\left(x\right)}\exp\left(\sum_{i,k}\lambda_{k}t_{k}\left(\lambda_{i+1},y_{i},x,i\right)-\sum_{i,k}\mu_{k}\rho_{k}\left(\mu_{i+1},\rho_{i},x,i\right)\right). \end{array}$$

In addition, in the process of concept relation extraction, relations that cannot be extracted directly are first extracted by image similarity, which in turn enables the calculation of the requested category relations. Finally, in the hierarchy building process, concept entropy is proposed and the resulting concept entropy is used to analyze the semantic breadth of the concept. The above model allows for the filtering and processing of noisy labels with a high redundancy [15]. The different categories of relations in multimodal data can be roughly classified into simultaneous and hierarchical relations, and in general, generic concepts are used more frequently than specific concepts, so the statistical phenomena of entity text data and image data features can be calculated based on this status quo in the process of relation extraction.

(2) $$\begin{array}{c} Z\left(x\right)=\exp\left(\sum_{i,k}\lambda_{k}t_{k}\left(\lambda_{i+1},y_{i},x,i\right)-\sum_{i,k}\mu_{k}\rho_{k}\left(\mu_{i-1},\rho_{i},x,i\right)\right). \end{array}$$

Due to the differences in the trading mechanism of each pilot carbon market and the different ways of accounting for carbon emissions in each pilot, there is not much trading liquidity among the pilots, which tends to form regional barriers. In addition, prices in the market are also relatively volatile, and without a unified regulator, carbon market trading still has certain risk potential, and the allocation of carbon emission allowances needs to seek a trade-off between fairness and efficiency. Therefore, there is a need to establish unified carbon emission rights trading rules, mechanism monitoring, management, assessment system, etc. A special coordination and forecasting organization shall be established to carry out unified planning and market dispatching for power generation, predict the risk of instability of water demand and power generation load machine, and predict the opacity of market information and the decline of carbon price. The government also faces the risk of unstable policy expectations in stimulating the carbon market. The opaque market information and the decline of carbon prices may affect the carbon market to promote the emission reduction function and regional enterprise synergy through scientific resource allocation and development. Stimulate market vitality, increase the transparency of the carbon market, regulate management, and improve the enthusiasm of enterprises to participate, etc.

(3) $$\begin{array}{c} \text{Accuracy}\left(D,I\right)=\frac{\sum_{i\in I,d\in D}\text{check}\left(d^{2},i^{2}\right)}{I}. \end{array}$$

**Figure 1:**
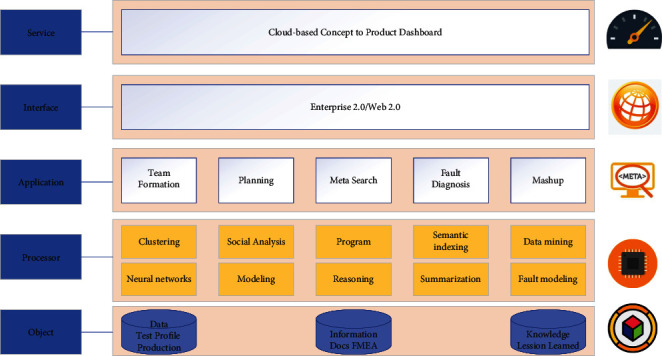
Knowledge mapping technology framework diagram.

Clarify the financial attributes of the carbon market, classify carbon quotas as financial instruments, and include carbon quota spots, derivatives, and other carbon financial products in financial regulation to avoid market risks, and maintain the stability of enterprises' participation in trading. As some enterprises must pay higher costs to complete compliance, some carbon markets are not actively trading, while carbon financial products can avoid risks, thus effectively reducing the compliance costs of the relevant enterprises and increasing the enthusiasm of enterprises to participate in carbon emission reduction [16]. At the same time, laws, regulations, and rules related to carbon emissions trading should be introduced to strengthen the binding force of carbon emissions trading and ensure the smooth implementation of carbon emissions trading. In addition, information on carbon emissions trading should be made public to enhance the transparency. Develop relevant mechanisms to attract public scrutiny, strengthen publicity, and enhance public awareness of energy-saving and emission reduction, to lay the foundation for the construction of a nationally unified carbon emission trading market, as shown in Figure 1.

The architecture contained in this paper is mainly divided into four core technologies, among which data acquisition mainly adopts methods and techniques such as crawling, downloading, and purchasing for data collection, and uses operations such as word separation, de-duplication, and de-noising to complete data processing; multimodal knowledge extraction mainly adopts machine learning methods to extract key information in the data; multimodal knowledge fusion requires disambiguation and conflict detection of knowledge to achieve graph visualization and upper-layer applications. In the process of establishing a carbon financial market and bringing in a wide range of investors from financial institutions to participate in the trading of carbon financial assets, it is also important to prevent excessive speculation in the market. In the case of physical commodities with real demand, a negative feedback mechanism will gradually be formed to “correct” the price when it deviates from the value, thus achieving a dynamic equilibrium where the price fluctuates up and down around the value.

The number of layers in an RNN is the number of layers in the network. Carbon assets can be traded in the carbon market and are given the attributes of a commodity. The investment nature of carbon assets is that they can be traded freely in an open and active carbon market and that the transaction generates an inflow of economic benefits for the company. This indicates that the carbon assets are investment properties of financial assets. Emission control companies actively engage in technological upgrading and equipment replacement to obtain surplus carbon dioxide credits, which are then traded in the carbon market in exchange for revenue, indicating that the carbon assets have a certain investment value.

(4) $$\begin{array}{c} P_{i}=f_{\text{pooling}}\left(M_{i}^{2}\right). \end{array}$$

Existing tests of higher-order moment risk contagion are usually based on event-based analysis, which examines the risk contagion of higher-order moment attributes between financial markets before and after exceptional or sudden major events, such as financial crises. On the one hand, this event-based risk contagion before-and-after segmentation approach conceals the heterogeneity of volatility trends in the financial market return series itself, and the volatility of carbon financial asset prices itself contains market information reflecting major unexpected events; on the other hand, it fails to comprehensively identify the triggering factors of risk contagion in carbon financial assets and implies an unreasonable assumption that financial crises or unexpected creation of factors that would lead to risk contagion in the carbon finance market.

(5) $$\begin{array}{c} E\left(R_{m}\right)=w_{i}E\left(R_{i}^{2}\right)-w_{i}E\left(r_{i}^{2}\right). \end{array}$$

**Figure 2:**
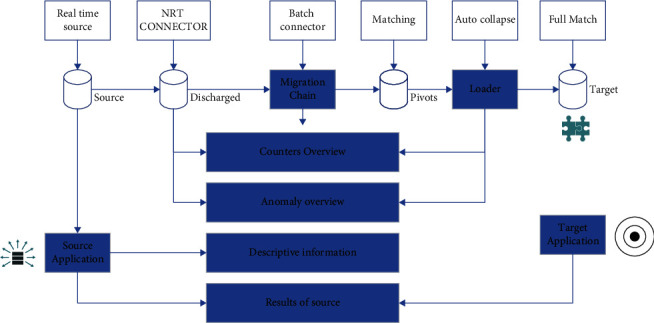
Multimodal data processing flow.

Therefore, the study of risk contagion of higher-order moment properties of carbon financial assets must be based on the special volatility trend heterogeneity characteristics of the carbon financial market, and uncover the risk contagion relationship between carbon prices and their pricing factors under different market volatility trends due to asymmetric market information and extreme event shocks, as well as the impact mechanism of this contagion relationship on carbon prices, as shown in Figure 2.

Among them, as the main interface of most websites nowadays displays the content of all image pages as thumbnails, which contain the address index of the original image, accessing the address of the original image requires accessing the index information of the thumbnail, and for the above characteristics that most web pages have, in this paper, in the process of image data crawling, the index page address of the original image is analyzed although it will be different because of the image, the change of this address. In this paper, we analyze the index page address of the original image [17]. Still, it varies from image to image, the address changes in a certain pattern, i.e., only the last data bit of the image changes. Using such characteristics of the image address, it is possible to obtain many image index page addresses based on a fixed address bit.

### 3.2. Analysis of Digital Neural Network Models

Among the existing knowledge extraction methods, the use of segmented convolutional neural networks for keyword extraction can improve the semantic degree of feature vector representation, reduce the sparsity of data dimensions, and effectively solve the problem of data noise. In addition, for the input text data, some words may contain more important semantic information than other parts, so introducing an attention mechanism to focus on key information and ignore irrelevant information can reduce the interference caused by irrelevant information to the semantic feature extraction and make the key information extracted by the model more accurate. Based on this, this paper uses a segmented convolutional neural network model that introduces an attention mechanism to extract the relationships implied in the data of people and events related to party building and to extract the important information in the original data. The original data is fed into the model for extraction along with the target entities to be extracted. Promote the application of green credit evaluation and management systems among banking institutions, require financial institutions to conduct environmental risk stress tests, strengthen the green credit statistical system, support regions in establishing green project banks, and effectively enhance the scale of green credit and the business capacity of green credit in the province. Encourage banks to issue green bonds and green asset-backed securities to support the construction of green projects. Study the establishment of a risk compensation mechanism for green loans and increase the flow of green credit to support small and medium-sized innovative enterprises. Encourage banking institutions to issue green financial products and spread public awareness of green development through investment products.

**Figure 3:**
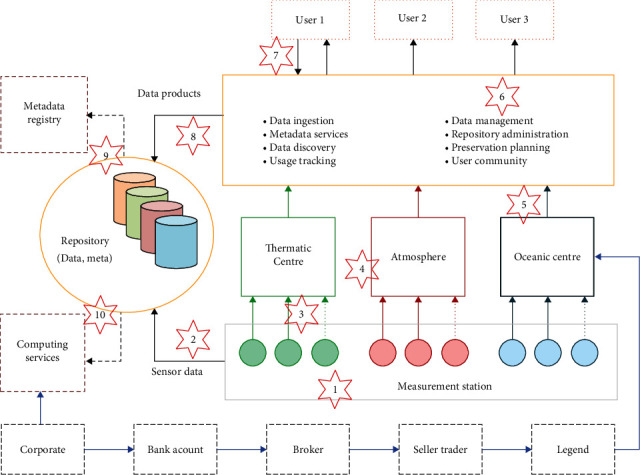
Data integration to be addressed.

The impact of carbon emissions trading on power generation enterprises also includes the following aspects. Firstly, the participation of power generation enterprises in carbon emissions trading will inevitably increase the cost of carbon allowance trading, technical transformation, and management costs. Secondly, it affects the production methods of enterprises [18]. Thermal power enterprises with difficulties in reducing emissions will inevitably consider adjusting their energy mix to avoid penalties or quota shortage situations and promote the low-carbon transformation of their energy mix. Accelerate the research and development of renewable energy generation, carbon capture, low-carbon, and efficient coal-fired gas technologies, and the transformation of solar photovoltaic grid-connected power generation technologies, as shown in Figure 3. Carbon trading, an important mechanism for using market economics to promote environmental protection, allows companies to use or trade energy from within the company as well as domestically and internationally with these reduced carbon emissions, provided that the total emissions specified in carbon trading are not exceeded.

Carbon emissions trading is both a challenge and an opportunity for the power generation industry. In the carbon emissions trading market, high-carbon enterprises are driven to transform to low-carbon by the cost of purchasing carbon allowances resulting in lower economic efficiency. At the same time, low-carbon enterprises will see increased economic benefits from the sale of excess carbon allowances, increasing investment in low-carbon industries, and improving the resource allocation efficiency. The inclusion of thermal power companies in the national carbon market and the implementation of emissions reductions will inevitably increase their costs. With the future tightening of quota allocation and reduction in the proportion of free quotas, this cost pressure will gradually increase, especially for some inefficient and backward small units, which may be accelerated and shut down. The dual carbon target will accelerate the construction of the national carbon market, fully explore carbon emission reduction (CCER) assets, and establish a sound carbon emission management system for the company. Emphasis will be placed on building the carbon management capacity of the team to grasp the initiative and gain a competitive advantage for the company when the national carbon emissions trading market is launched. The implementation of carbon trading may initially put pressure on companies to reduce emissions costs, but in the long term, if companies are proactive and innovative and adopt appropriate strategies, they will reap more benefits from the carbon trading market.

**Figure 4:**
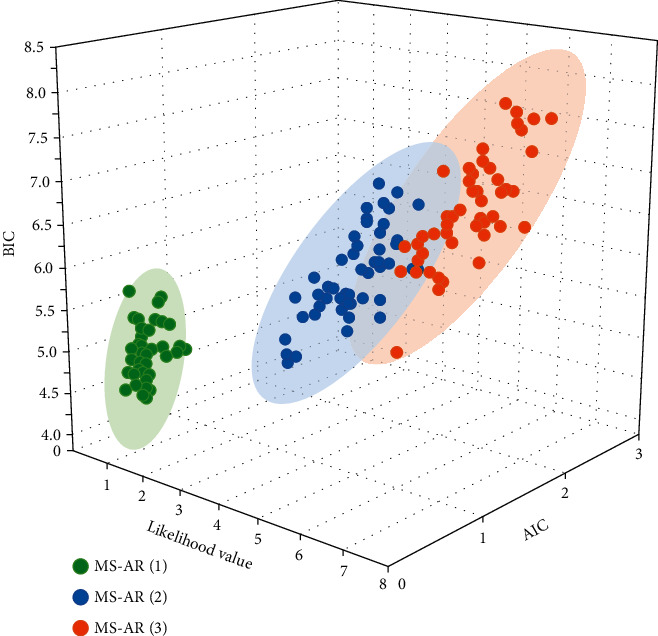
Parameter estimation of the volatile state transition model for carbon financial asset markets.

The entity tags associated with imaginative visuals and the entity tags associated with image descriptions are first associated with WordNet resources and DBpedia resources, respectively [19]. This ensures good scalability of the data. Based on the hierarchy defined by WordNet and DBpedia, it is possible to extend the vocabulary with more semantically related contextual terms that are associated with the images, which plays a key role in both calculating image correlation and enhancing the relevance of the data, as shown in Figure 4.

The analysis of the mechanism shows that carbon trading volume and carbon price affect the carbon abatement cost, so enterprises need to control the carbon trading volume to reduce the abatement cost. The amount of coal used and the research of energy-saving technology affect carbon emissions and thus carbon trading volume. Therefore, enterprises should carry out scientific and technological innovation, research, and development of carbon-saving technology, and control the amount of coal used to influence carbon trading volume and thus carbon abatement costs. The price of carbon is indirectly caused by carbon emissions and is influenced by both the price of coal and the volume of carbon traded. Carbon trading volumes and carbon prices are key variables in the system cycle. As carbon trading volumes can be estimated based on historical data, fluctuations in carbon prices will cause changes in the cost of carbon emissions, completing the impact on the economic efficiency of the business. Similarly, when the carbon price is fixed, the change in carbon trading volume will also have a corresponding effect [20]. The feedback mechanism can encourage emission control enterprises to store allowances when the carbon price is low, to minimize the cost of compliance; it can also encourage emission control enterprises to sell surplus allowances for profit when the carbon price is high, to improve the economic efficiency of enterprises. The carbon footprint is used as an important indicator to evaluate the greenness of projects, the greenness of financial institutions, and the strength of policy support. New technologies and methods can also be used, such as joint collaboration among the government, enterprises, financial institutions, and industry associations to build a big data platform for enterprise carbon emissions and environmental information, and encourage third-party organisations to develop public products of data using technologies such as satellite remote sensing and blockchain to provide enterprises and financial institutions with refined management tools for risk prevention and control. Based on this core concept, the model is constructed using scenarios to control variables and reflect the effects of changes in the carbon market.

**Figure 5:**
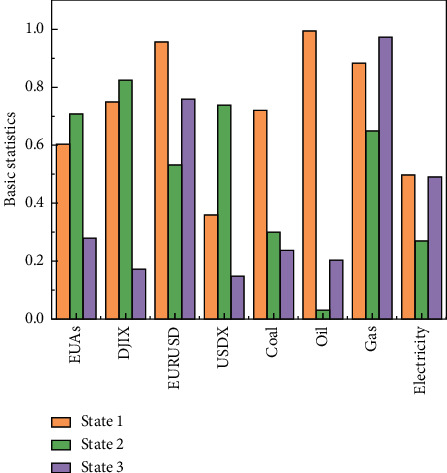
Basic statistics of the co-high order moments of a carbon financial asset and its pricing factor.

The standard deviations of the three states were found to be 1.17%, 6.94%, and 2.39%, respectively, which are defined as stable, high, and low volatility according to the relative size of the standard deviation. An analysis of the standard deviation of each state shows that the standard deviation of high volatility is equivalent to the three times that of low volatility, six times that of stable volatility, and two times that of stable volatility, as shown in Figure 5.

Indicates that the carbon financial asset has a right-skewed distribution relative to its pricing factor and there is a greater scope for profit-taking in the market; conversely when the degree of co-skewness is less than 0, it indicates that the carbon financial asset has a left-skewed division relative to the pricing factor and the probability of market downside increases. Curtness is a measure of the relative kurtosis of the two assets, where Curtness 13 represents the relative kurtosis of carbon financial asset returns compared to the pricing factor and Curtness 31 represents the relative kurtosis of the pricing factor compared to the carbon financial asset. A larger Curtness 13 means that carbon financial asset returns are more susceptible to extreme events compared to the pricing factor market. Conversely, the price of carbon financial assets is relatively stable.

## 4. Analysis of Results

### 4.1. Analysis of Numerical Multimodal Knowledge Mapping Performance Results

The main function of factor analysis is to extract the variables with strong correlation based on all the information that has been input, and to construct a common factor by extracting the common information of the variables, to judge the feasibility of building a factor analysis model. If there is strong independence between the input variables, or if the input variables are weakly correlated, then it can be assumed that there is less public information between the input variables, so it is difficult to achieve the desired results using factor analysis methods. The current multivariate checking methods include Bartlett's spherical test and the KMO test, which test the correlation between the initial input variables, and the commonality test, which tests the initial input variables by one variable. If the correlation containing the tested variables is significant, then a high degree of correlation between the input multiple variables is considered and factor analysis can be used. Information extraction techniques that integrate multiple modalities are the basis for constructing multimodal knowledge bases that can provide sufficient data support for many applications such as question and answer systems and information retrieval, so there is a great potential for development and application in this research direction. However, multimodal research also presents great challenges, with duplication and noise in the information between different modalities, and how to solve these problems is the key to handling the multimodal tasks. Before the input data is tested, the multivariate is standardized, which in turn removes the influence of the magnitude in the initial variables.

**Figure 6:**
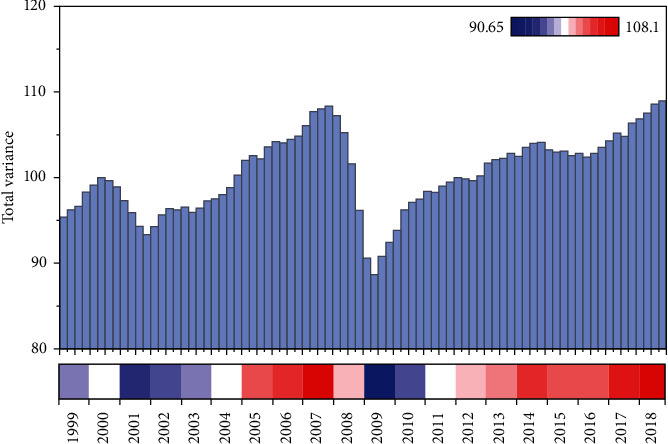
Total variance explained.

A total of four public factors have a cumulative contribution of 80% or more and the results of the characteristic root variables are greater than 1. Therefore, these four public factors can be used as the classification of indicators affecting the formation of carbon trading price risk, namely, *F*1, *F*2, *F*3, and *F*4, with a cumulative contribution of 87.162% of the four public factors, indicating that these four factors can be used as explanatory factors affecting the carbon trading price risk; the four common factors are used as explanatory variables for the price risk of carbon trading. After rotating the factors, using the maximum rotation method, the variance contribution of the four factors was obtained as 36.986%, 63.464%, 75.678%, and 87.162% respectively. These four public factors alone can contain all the information of the variables, so the article has taken these four factors with a good ability to explain the raw data as the public factors, as shown in Figure 6.

Exploring the issue of small proportional carbon allowance allocation in the context of carbon emissions trading can effectively understand the factors affecting the cost of carbon emission reduction and the proportion of enterprise cost composition, to reduce compliance costs and improve enterprise efficiency for power generation enterprises in the light of the actual situation. It also provides a scientific reference for the allocation of allowances in China's national carbon emissions trading market. A reasonable range of carbon quota allocation schemes can ensure the efficiency of enterprises while increasing the enthusiasm of power generation enterprises to participate in carbon emission reduction and further enhance the enthusiasm for carbon verification work. A small percentage of carbon quotas can be dynamically adjusted to respond effectively to changes in the carbon trading market environment and national policies, with the expectation that the implementation of the carbon trading mechanism will positively promote corporate reform and innovation, ensuring that the companies will improve their economic efficiency while adjusting their energy mix to practice low-carbon development and actively respond to the business survival environment. The allocation of carbon emission allowances is the most closely related aspect of the design of a carbon trading system. After the establishment of the carbon emissions trading system, the scarcity of allowances will form the market price, so the allocation of allowances is essentially the allocation of property rights, and the way in which allowances are allocated determines the cost for enterprises to participate in the carbon emissions trading system.

**Figure 7:**
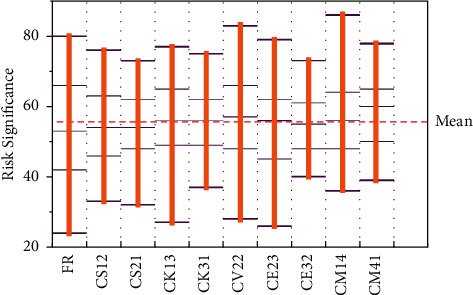
Statistical significance of risk contagion channels.

Comparing the intensity of risk contagion during the slow rise and slow fall of carbon price volatility, the intensity of risk contagion during the slow rise trend is generally higher than that of risk contagion during the fall trend, a finding that is largely consistent with the difference in the intensity of risk contagion during the rapid fluctuation trend of the carbon price. A slow decline in carbon prices indicates a slow reduction in the market risk of carbon financial assets or pricing factors, a lower level of risk, and a lower probability of sharp fluctuations in returns due to the hidden extreme event shocks in market prices, and thus a weaker risk contagion intensity, while a slow rise in carbon price volatility and an increase in carbon price risk indicates a gradual increase in carbon price risk and pricing factor risk, corresponding to a stronger risk contagion intensity, as shown in Figure 7.

These products and channels of risk contagion with carbon financial assets are integrated into the theoretical framework of multi-factor pricing of carbon financial assets with higher-order moment attributes, thus forming the analytical basis for measuring the pricing framework of carbon financial assets considering risk contagion with higher-order moment attributes proposed in this paper. Under the impetus of China's dual carbon targets, the total amount of carbon quotas in the domestic carbon trading market will be tightened in the future and it will become more difficult for power companies to obtain free carbon quotas, with a portion of the quotas inevitably being allocated to companies for a fee by way of auction. Once thermal power enterprises need to purchase quotas by way of auction, this part of the cost will be directly internalised and push up the cost of electricity for the enterprises. The finding that the intensity of risk contagion is higher under the trend of rapid market volatility than the trend of slow market volatility is not only consistent with the heterogeneous nature of carbon financial asset price volatility, but also portrays the asymmetric nature of carbon financial asset prices due to volatility heterogeneity.

### 4.2. Analysis of Numerical Neural Network Model Results

The number of iterations represents the number of times the model parameters are updated, and the appropriate number of iterations has a significant impact on the output of the neural network. The acceptable training loss of the model is usually taken as the target, and the optimal number of iterations is the number of times the model is trained to achieve this target loss. It is found that the carbon financial asset pricing framework considering higher-order moment risk contagion can approach the optimal target loss within 2000 iterations, i.e., the model loss is stable through 2000 parameter updates and gradient adjustments, and convergence of the model structure can be achieved. The Ministry of Ecology and Environment shall, in accordance with national greenhouse gas emission control requirements and taking into account factors such as economic growth, industrial restructuring, optimization of the energy structure and synergistic control of air pollutant emissions, formulate a plan for determining and allocating the total amount of carbon emission quotas. Provincial ecological and environmental authorities shall allocate carbon emission quotas to key emission units within their administrative areas in accordance with the plan for determining and allocating the total amount of carbon emission quotas formulated by the Ministry of Ecology and Environment.

**Figure 8:**
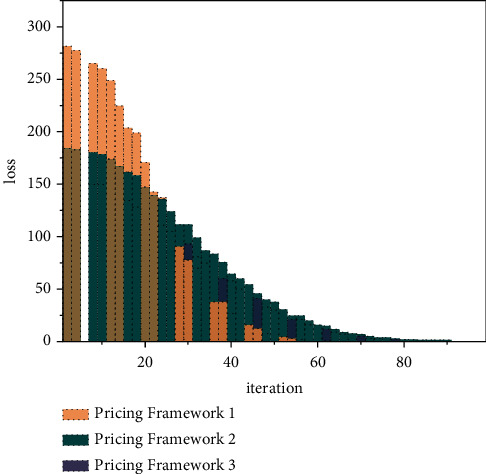
Convergence of model iterations for different pricing frameworks.

The Pricing Framework, a pricing framework for carbon financial assets that considers the risk contagion relationship for higher-order moment attributes, has higher efficiency and shorter model convergence over 2000 iterations. In contrast, the other pricing frameworks do not converge effectively or have large errors within 2000 iterations, and in terms of efficiency, more time is needed for learning and training. Therefore, it is reasonable to set the training of the network structure within 2000 iterations, as shown in Figure 8.

The learning rate of machine learning determines the speed of updating the model parameters and the convergence of iterations of the optimizer. The traditional gradient descent algorithm uses a fixed step learning rate which may lead to model training that does not effectively approximate the optimal solution, resulting in weak model generalization. In this paper, the adaptive moment estimation Adam algorithm can not only update the model parameters according to the differences in the dimensionality of the data moment attributes, i.e., a larger update for the low-frequency parameters and a smaller update for the high-frequency data, but also determine the rationalization range of the parameter update through the momentum adjustment of the moment attributes, and optimize the model according to the dynamic learning rate, which enables the model to have better robustness. Key information is a prerequisite for the implementation of scientific command decisions. The key information needs of commanders will directly affect operational decision-making and the smooth implementation of military operations. By establishing information priority levels and clarifying the focus of information acquisition, the command structure's attention is always focused on high-value information, and the key information to support combat decision-making is quickly mined from the massive, multiple, and heterogeneous intelligence information to identify intentions, discover signs, study and judge trends, and match the best action strategy, which can help commanders effectively compress the time cost of the observation, adjustment, decision, and action. It can help commanders to effectively reduce the time cost of the “observation, adjustment, decision, and action” cycle.

When the initial hyperparameter learning rate of the adaptive moment estimation Adam algorithm is 0.003, the network training has the smallest training error of 0.009394, i.e., the average training error decreases gradually with the increase of the learning rate, and drops to the lowest point at the learning rate of 0.003, and then the average error gradually increases with the increase of the learning rate, so the initial learning rate is set to 0.003 in this paper. Further, the analysis of the loss error for different learning rates within a given iteration range shows that when the initial hyperparameter learning rate is 0.003, the model not only converges effectively to the given objective function value but also has low training error fluctuations.

**Figure 9:**
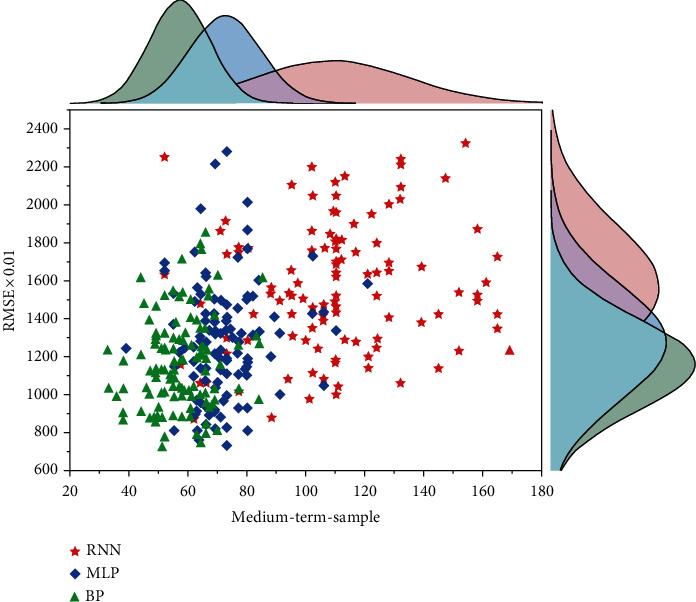
Prediction effect of the medium-term fitting term-based carbon financial asset pricing framework.

The accuracy-recall curve of EDDSRE lies at the top of the three modified versions. In terms of accuracy, at a recall equal to 0.5, the accuracy improvement of EDDSRE is greater than 20% compared to EDDSRE-ATT-IQ-PartialMax; in terms of recall, compared to EDDSRE-ATT-IQ-PartialMax, the EDDSRE has a recall improvement of greater than 10%. This suggests that the use of an attention mechanism to dynamically adjust the package representation to highlight the features of the predicted relations, the use of information to determine the training order of the relations in the training instances, and the use of partial maximum pooling to initialize the package representation to alleviate the mislabeling problem of remote supervision, can gradually improve the accuracy and recall of the relation extraction, as shown in Figure 9.

Based on the estimated errors for the different maturity pricing frameworks, it is found that for the Pricing Framework, as the maturity gradually increases from short-term to long-term and the forecast sample time increases, all the forecast errors of the forecasting models gradually decrease, indicating the improvement of model accuracy and stability. In particular, the Multi-LSTM model has the smallest forecast error values and the best forecasting results, i.e., the short-term forecast errors RMSE, MAE, and MAPE are 1.804855, 1.458599, and 1.577122, respectively, while the medium-term forecast errors RMSE, MAE, and MAPE are 1.487359, 1.204297, and 1.047486 respectively. Long-term forecast errors RMSE, MAE, and MAPE are 1.343231, 1.102491, and 0.943605, respectively. The forecast accuracy and stability of the Multi-LSTM model are gradually optimized as the forecast time is extended and significantly outperforms other benchmark models, enabling effective forecasting of 12-month lagged returns. As the LSTM has the advantage of being good at fitting and forecasting data with longer time series, the conclusions of this paper provide further evidence to support this.

## 5. Conclusion

An entity linking method that combines a common attention mechanism with a graph convolutional neural network has been proposed for entity linking for knowledge graphs. Existing neural network-based entity linking methods ignore the semantic gap between the context of sequential entity denotative items and the context of graph-structured entities, thus affecting the entity chain by *N* any-add rate. It is observed that the importance of each word in the entity denotative item context is different, as is the importance of the content in the entity context. To solve the above problems, this paper proposes an entity linking method that combines a common attention mechanism with a graph convolutional neural network. The method uses the common attention mechanism to establish the correlation between the context of entity referent items and the entity context to narrow the semantic gap between them and model the importance of the content in both at the same time. Based on this, a context-aware graph convolutional neural network is proposed for learning the representation vectors of entities, considering the significant graph topology features of entity contexts. Experiments were carried out on five publicly available datasets, and the experimental results show that the entity linking method combining the common attention mechanism with the graph convolutional neural network has higher accuracy, recall, and *F*1 values than the comparison method.

## Data Availability

The data used to support the findings of this study are available from the corresponding author upon request.
